# Associations of lifestyle and biological factors with body image among reproductive-aged women: a cross-sectional study

**DOI:** 10.1038/s41598-026-56798-7

**Published:** 2026-06-19

**Authors:** Olívia Dózsa-Juhász, Viktória Prémusz, Pongrác Ács, Márta Hock, Alexandra Makai

**Affiliations:** 1 Doctoral School of Health Sciences, Faculty of Health Sciences, University of Pécs, Pécs, Hungary; 2 Institute of Physiotherapy and Sport Science, Faculty of Health Sciences, University of Pécs, Pécs, Hungary; 3 Physical Activity Research Group, Szentagothai Research Centre, University of Pécs, Pécs, Hungary

**Keywords:** 24-hour movement behaviours, Body image, Premenstrual syndrome, Sleep quality, Women’s health, Diseases, Health care, Medical research, Risk factors

## Abstract

This study aimed to identify lifestyle and biological factors associated with body image among reproductive-aged women, focusing on physical activity, sedentary time, sleep, BMI, premenstrual symptoms (PMS), and sleep quality. A secondary aim was to explore interrelationships among these factors to contextualise their combined association with body image. In this cross-sectional online survey, 291 women aged 18–45 years participated (mean age: 27.16 ± 0.36 years). Validated questionnaires were used to assess body image (Body Appreciation Scale), physical activity (Global Physical Activity Questionnaire), sleep quality (Sleep Quality Scale), BMI, and PMS (Premenstrual Assessment Form-Short Form). Data were analysed with IBM SPSS 28.0 using multivariable linear regression and correlation analyses (*p* < 0.05). Higher BMI, greater PMS severity, poorer sleep quality and longer sedentary time were significantly associated with lower body appreciation. Correlations were found between PMS and sleep quality (*r* = 0.309; *p* < 0.001), PMS and work-related moderate activity (*r* = 0.119; *p* = 0.043), and sleep quality and work-related moderate activity (*r* = 0.169; *p* = 0.004). No significant associations were observed for recreational physical activity with PMS (*r*=-0.041; *p* = 0.544) or sleep quality (*r*=-0.040; *p* = 0.498). Body image in reproductive-aged women was associated with both lifestyle behaviours and biological symptom burden. The findings suggest that supporting positive body image may benefit from integrated approaches addressing sedentary behaviour, sleep quality and menstrual health alongside weight-related factors.

## Introduction

Body image is a multidimensional concept that encompasses self-perception and associated attitudes, including feelings, thoughts, and behaviours related to one’s own body^1^. These body image attitudes are associated with self-esteem, interpersonal confidence, eating and exercise habits, grooming activities, sexual behaviours and experiences, as well as emotional stability. Factors affecting body image can range from positive to negative, making it crucial to examine the determinants of body image^2^.

Body image concerns are particularly prevalent among women of reproductive age, a period characterized by heightened sociocultural appearance pressures, hormonal fluctuations, and changing health behaviours. Previous studies indicate that body dissatisfaction is more frequent in young adult women than in men and is strongly associated with psychosocial stressors and health-related behaviours^3,4^. These factors may render body image especially sensitive to both lifestyle patterns and biological symptom burden in this population.

Individuals with negative body image may suffer from body image disorders. Body image disorders can be a source of various health issues, including eating disorders^5^, susceptibility to depression^4^, deteriorating physical and mental health^6^, or the initiation of smoking^7^.

Body image is influenced not only by anthropometric characteristics but also by lifestyle behaviours and physiological factors. Lifestyle patterns such as sedentary behaviour, physical activity, and sleep are increasingly recognised as interconnected components of 24-hour movement behaviours, which together may influence both physical and psychological aspects of health^8–12^. At the same time, premenstrual symptoms represent a common physiological experience among women of reproductive age and may also be associated with sleep disturbances, mood changes, and body perception^13–15^. Understanding how these behavioural and physiological factors jointly relate to body image may therefore provide a more comprehensive perspective on women’s health.

The primary aim of the present study was to identify lifestyle and biological factors associated with body image among reproductive-aged women, with particular emphasis on physical activity, sedentary behaviour, body mass index, premenstrual symptom severity and sleep quality. A secondary aim was to explore the interrelationships among these factors in order to better contextualise their combined influence on body image.

## Materials and methods

The present cross-sectional study was conducted between September and October 2023. Convenience sampling was used to select participants for the study. Using the Google Forms application, we created an open, self-administered questionnaire, which we distributed to potential participants through women’s health-themed social media groups, reaching approximately 1500 individuals. The invitation description for the questionnaire included the online link to the survey, the objectives of the research, as well as a consent form and patient information sheet to be completed. The research included women aged between 18 and 45 years who are native Hungarian speakers and provided consent to participate in the study based on the inclusion criteria. Participants who were pregnant, breastfeeding, or diagnosed with early ovarian insufficiency at the time of filling out the questionnaire were excluded from the study.

The questionnaire underwent a preliminary testing phase involving 30 participants, following which minor adjustments, primarily addressing linguistic nuances, were made before finalisation and distribution. The completion of the questionnaire was not mandatory, and every respondent voluntarily consented to participate. Furthermore, no compensation or incentive was offered in exchange for participation. The final version of the questionnaire consisted of 3–4 questions per page, totaling 41 pages. By employing adaptive questioning, irrelevant questions for individual participants could be skipped without interrupting the completion process. During the completion process, participants had the opportunity to review and modify their answers. No cookies were used on the questionnaire website. The respondents remained anonymous throughout (email addresses were not collected) and were assigned unique timestamps to avoid duplication. The anonymous nature of the survey and the absence of personally identifiable information were intended to reduce potential social desirability bias. During the preliminary testing, a minimum time limit of 7 min for completion was established. Questionnaires completed in less time than this threshold were excluded. Only fully completed questionnaires were eligible for final submission.

The first part of the questionnaire contained socio-demographic, anthropometric, general health, and women’s health-related questions. Additionally, it surveyed the average weekday and weekend sleep duration (in hours) with the following questions used: “Please indicate how many hours you sleep on average on weekdays and weekends!”. Participants were able to give their answers in hours. The second part of the questionnaire consisted of separate, validated surveys aligned with the research objectives.

Participants’ subjective body image was measured using the Hungarian validated version of the Body Appreciation Scale (BAS)^16,17^. The BAS questionnaire is used to assess positive body image. The questionnaire comprises 13 questions within 4 subscales, to which participants respond using a 5-point Likert scale (1–5). The lowest possible score achievable is 13 points, while the highest is 65 points. The higher the score someone achieves, the more positive their own body image can be considered.

Physical activity was assessed using the Hungarian validated version of the Global Physical Activity Questionnaire (GPAQ)^18^. WHO developed the Global Physical Activity Questionnaire for physical activity surveillance in countries. It collects information on physical activity participation in three settings (or domains) as well as sedentary behaviour, comprising 16 questions. The domains are: Activity at work; Travel to and from places; Recreational activities. The results of physical activity were presented in minutes per week, hours per day, or minutes per day. For analysis purposes these domains can be further broken down into six different “sub-domains”. These “sub-domains” are: Vigorous work; Moderate work; Travel; Vigorous recreation; Moderate recreation and Sitting.

We used the Hungarian validated version of the Premenstrual Assessment Form-Short Form (PAF-SF) questionnaire to assess premenstrual symptoms^19,20^. The PAF-SF is a ten-item questionnaire developed to provide a detailed description of premenstrual changes affecting both mood and physical condition. The questionnaire evaluates these changes through three subscales: (1) “Pain,” (2) “Water Retention,” and (3) “Affect.“. The Affect subscale comprises four questions, while the Water Retention and Pain subscales each contain three questions. Answers are recorded on a six-point scale ranging from 1 (no change) to 6 (extreme change). The lowest achievable score is 10, while the highest is 60 points. A higher score indicates more severe premenstrual symptoms. The questions pertain to the symptoms experienced during the last three menstrual cycles.

We utilized the Hungarian-translated version of the Sleep Quality Scale (SQS) questionnaire to assess sleep quality^21,22^. The SQS is a questionnaire comprising 28 questions that measure participants’ subjective sleep quality through 6 subscales. Respondents can assess their own sleep quality using a 4-point Likert scale (0–3), with the lowest possible score being 0 points and the highest possible score being 84 points. The result is obtained based on the total score. The higher the total score, the more severe the assumed sleep quality problem.

Participants’ BMI was calculated based on self-reported weight and height, using the standard BMI formula (weight in kg/height in m²), and classified according to the five BMI categories defined by the WHO^23^.

The study was registered by the U.S. National Institute of Health (NCT06146673||https://www.clinicaltrials.gov/) with the Clinical Trial Registry (Registration Date: 11/23/2023)) and approved by the Medical Research Council of Hungary (Identifier: BM/23899-1/2023).

A research protocol adhering to the principles of the Helsinki Declaration was followed^24^. Participants provided voluntary, uninfluenced informed consent for the anonymous use and publication of their data following appropriate information provision.

The current study was conducted following the guidelines of the Strengthening the Reporting of Observational Studies in Epidemiology (STROBE), the Checklist for Reporting Results of Internet E-Surveys (CHERRIES) and the Sex and Gender Equity in Research (SAGER) guidelines.

### Statistical methodology

During the research, we used Microsoft Office Excel 2016 for database creation and questionnaire evaluation. For statistical calculations, we utilised IBM SPSS version 28.0 (SPSS Inc., Chicago, IL, USA). Descriptive statistics included mean and standard deviation for scales. To assess the normality of the data distribution, we used the Kolmogorov-Smirnov normality test. To test the associations between the continuous variables, we employed Pearson correlation analysis for normally distributed data and Spearman’s rank correlation for not normally distributed data, as well as multivariate linear regression models (outcome variable: body image; explanatory variables: BMI, premenstrual symptoms, sleep quality and time, time spent sitting, time spent with recreational activities). Prior to interpreting the regression model, the assumptions of linear regression were examined. Residual plots were inspected to assess linearity and homoscedasticity, while the distribution of standardised residuals was evaluated for approximate normality. Independence of residuals was assessed using the Durbin–Watson statistic. No major violations of regression assumptions were detected; therefore, the regression model was considered appropriate for interpretation. Correlation magnitudes were interpreted according to Cohen’s conventional criteria^25^, and 95% confidence intervals for correlation coefficients were calculated using Fisher’s r-to-z transformation. The adequacy of the sample size for the multiple linear regression model was evaluated based on conventional power considerations using G*Power 3.1 software^26^. Assuming a medium effect size (f² = 0.15), an alpha level of 0.05, statistical power of 0.80, and six predictors in the model, the minimum required sample size would be approximately 98 participants according to standard power analysis procedures. The present study included 291 participants, which substantially exceeds this requirement and therefore provides sufficient statistical power to detect medium-sized effects. Although some variables showed minor deviations from normality, parametric statistics were retained due to the relatively large sample size and the robustness of linear regression models under such conditions. The significance level was set at *p* < 0.05.

## Results

**Fig. 1:**
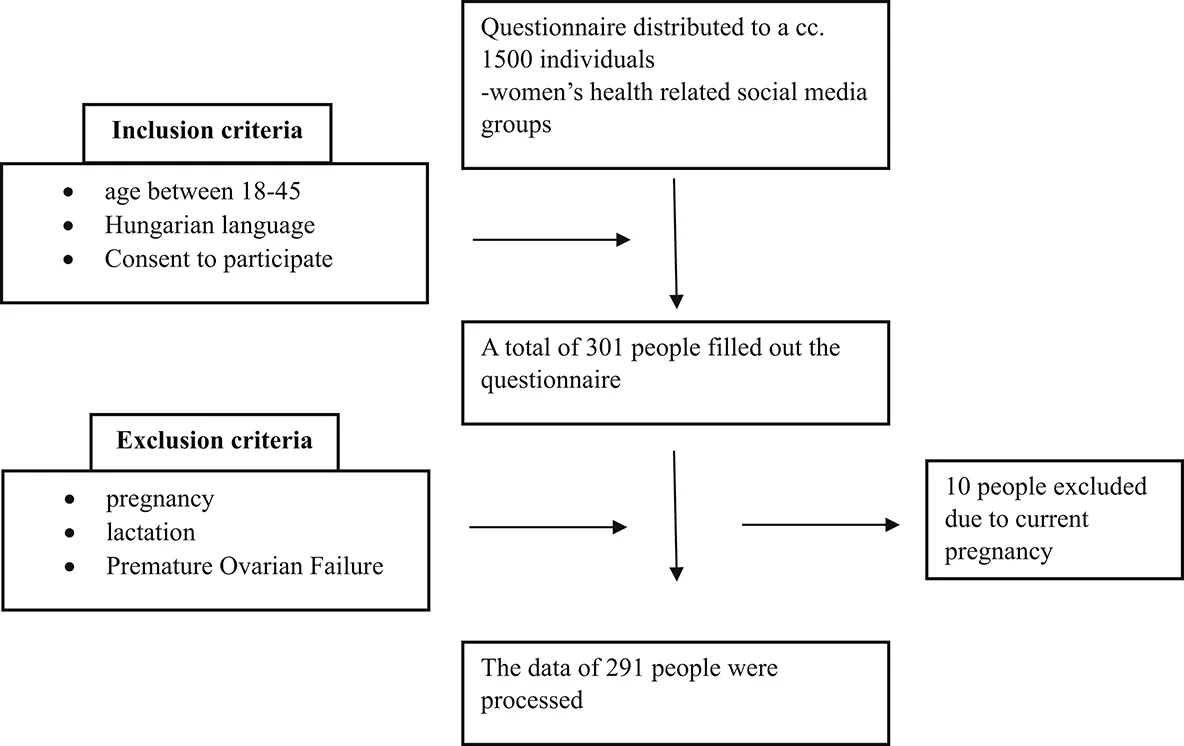
Flowchart representing the sampling process.

After reaching out to approximately 1500 individuals, a total of 301 individuals fully completed the questionnaire and gave consent to participate. Among them, 10 individuals indicated that they were pregnant at the time of filling out the questionnaire and were consequently excluded from the study according to the exclusion criteria. Thus, data from 291 individuals were analysed (Fig. 1.). The relatively low completion rate may be partly explained by the length of the questionnaire (41 pages), the voluntary nature of participation, the absence of financial or other incentives, and the inclusion of only fully completed questionnaires in the final analysis.

The descriptive characteristics of the sample are illustrated in Table 1.

**Table 1:** Descriptive characteristics of the sample (*N* = 291).

Characteristics	Mean ± SD
Age	27.16 ± 6.10 years
Height	167.11 ± 6.58 cm
Weight	63.62 ± 11.57 kg
Body mass index	22.77 ± 3.93 kg/m^2^
Sleep duration on workdays	7.01 ± 0.92 h/day
Sleep duration on the weekend	8.39 ± 1.17 h/day

The descriptive results of the validated questionnaires used in the study are intended to be presented in Table 2.

**Table 2:** The table illustrating the descriptive results of the validated measurement tools used in the study (*N* = 291).

Name of the questionnaire	Results
**Global Physical Activity Questionnaire**	
-vigorous work and moderate work	532.10 ± 802.87 min/week; *(1.77 h/workday)*
-vigorous recreation and moderate recreation	206.00 ± 455.44 min/week; *(29.24 min/day)*
-travel	491.24 ± 912.72 min/week *(1.16 h/day)*
-sitting	3015.56 ± 1428.86 min/week *(7.17 h/day)*
**Body Appreciation Scale**	44.64 ± 10.99 points
**Premenstrual Assessment Form-Short Form**	29.50 ± 9.57 points
**Sleep Quality Scale**	35.23 ± 12.55 points

Given the central role of body image as the primary outcome, additional correlation analyses were conducted to contextualise the regression findings and to explore potential background mechanisms among lifestyle and biological variables.

There was a positive significant correlation between sleep quality and the severity of premenstrual symptoms (*r* = 0.309**, 95% CI 0.202 to 0.409, *p* < 0.001). Furthermore, moderate work was correlated with sleep quality (*r* = 0.169*, 95% CI 0.055 to 0.279, *p* = 0.004), as well as moderate work and the severity of premenstrual symptoms (*r* = 0.119*, 95% CI 0.004 to 0.231, *p* = 0.043).

There was a negative correlation between weekday sleep duration and the following variables: total minutes per week in the Global Physical Activity Questionnaire (GPAQ) (*r*=−0.250**, 95% CI −0.354 to −0.139, *p* < 0.001), vigorous work (*r*=−0.179**, 95% CI −0.288 to −0.065, *p* = 0.002), moderate work (*r*=−0.134*, 95% CI −0.245 to −0.019, *p* = 0.022), vigorous recreation (*r*=−0.080, 95% CI −0.193 to 0.035, *p* = 0.171), moderate recreation (*r*=−0.080, 95% CI −0.193 to 0.035, *p* = 0.171), and travel (*r*=−0.266*, 95% CI −0.370 to −0.156, *p* < 0.001).

The Spearman correlation analysis results also revealed a negative correlation between average weekday sleep duration and sleep quality (*r*=−0.298**, 95% CI −0.399 to −0.190, *p* < 0.001), as well as between average weekday sleep duration and the severity of premenstrual symptoms (*r*=−0.043, 95% CI −0.157 to 0.072, *p* = 0.462).

There was also a negative correlation between vigorous recreation and sleep quality (*r*=−0.040, 95% CI −0.154 to 0.075, *p* = 0.498), as well as between moderate recreation and the severity of PMS symptoms (*r*=−0.040, 95% CI −0.154 to 0.075, *p* = 0.498).

In our research, we examined the factors affecting body image, the elements of 24-hour movement behaviour (physical activity and inactivity and sleep), the body mass index and the severity of premenstrual symptoms. The correlation results are illustrated in Table 3.

**Table 3:** The table illustrating the results of the correlation analysis regarding body image (*N* = 291).

Predictors	Correlation coefficient (*r*)	95% CI	*p*-value
BMI	−0.336	−0.434 to −0.230	< 0.001
PMS	−0.265	−0.369 to −0.155	< 0.001
Sitting	−0.169	−0.279 to −0.055	0.004
Recreational physical activity (vigorous and moderate)	0.138	0.023 to 0.249	0.018
Sleep duration	0.091	−0.024 to 0.204	0.120
Sleep Quality	−0.274	−0.377 to −0.164	< 0.001

Using conventional benchmarks for correlation magnitude, the association between BMI and body image was moderate, whereas the associations involving PMS severity, sleep quality, sedentary time, and recreational physical activity were small. The association between sleep duration and body image was negligible and non-significant. However, given the width of several confidence intervals, the magnitude and robustness of these bivariate associations should be interpreted cautiously. The effect of the explanatory variables on body image were examined using a multivariable linear regression model. The multiple linear regression model was statistically significant (R² = 0.238, F = 22.321, *p* < 0.001). Body mass index, premenstrual symptoms measured by the PAF-SF, sleep quality, and time spent sitting were significantly associated with body image, while recreational physical activity and sleep duration were excluded from the final model. (Table 4)

**Table 4:** Multiple linear regression model examining associations between lifestyle factors, premenstrual symptoms, sleep parameters and body image (*N* = 291).

Predictor	B	SE	Beta	t	*p*	95% CI Lower	95% CI Upper	VIF
Constant	80.22	3.93	–	20.41	< 0.001	72.48	87.96	–
Body mass index (BMI)	−0.83	0.15	−0.30	−5.69	< 0.001	−1.12	−0.54	1.02
Premenstrual symptoms (PAF score)	−0.25	0.06	−0.22	−3.96	< 0.001	−0.37	−0.12	1.13
Sleep quality (SQS score)	−0.17	0.05	−0.20	−3.57	< 0.001	−0.27	−0.08	1.13
Time spent sitting (minutes/day)	−0.001	< 0.001	−0.14	−2.69	0.008	−0.002	<−0.001	1.02

Model statistics: R2 = 0.238, Adjusted R² = 0.227, F = 22.321, *p* < 0.001, Durbin–Watson = 2.15.

Recreational physical activity and sleep duration were initially entered into the regression model but were excluded from the final model because they did not significantly contribute to the explained variance.

## Discussion and Conclusions

The present study aimed to identify lifestyle and biological factors associated with body image among reproductive-aged women, while secondary analyses explored interrelationships among these factors to contextualise their combined influence.

The mean Body Appreciation Scale score observed in the present sample indicates a moderate level of positive body image. This value appears comparable to those reported in previous European studies involving young adult women, suggesting that the examined population does not exhibit exceptionally high or low body appreciation^3,17^. Nevertheless, the identified associations highlight that even within normative ranges, body image remains sensitive to lifestyle behaviours and biological symptom burden.

During the research, we sought to identify factors that may be associated with variations in positive or negative body image. Based on the obtained results, our multivariable linear regression model identified several variables that were significantly associated with body image. These factors include body mass index, sitting, sleep quality, and the severity of premenstrual syndrome symptoms. The final regression model explained 23.8% of the variance in body image. This indicates a meaningful, but not exhaustive, proportion of explained variance, which is consistent with the multidimensional nature of body image. Comparable cross-sectional studies have reported varying proportions of explained variance depending on the outcome measure, sample characteristics, and predictor set. For example, Robbins et al., reported that their hierarchical regression model explained approximately 34% of the variance in body image discrepancy among adolescent girls, whereas Lee and Lee reported a model explaining 12% of the variance in body appreciation among adult Korean women^27,28^. In this context, the explanatory power of the present model appears broadly consistent with previous cross-sectional body image research, while also indicating that additional psychological, social, and contextual factors not assessed in the present study likely contribute to body image. BMI showed the strongest bivariate association with body image and was the only correlation reaching a moderate magnitude. Nevertheless, given the confidence intervals around the correlation estimates, these differences in magnitude should be interpreted with caution rather than as definitive evidence that weight-related factors are more robustly associated with body image than the other examined correlates. Currently, there is no available data in the existing literature to collectively support the validation of these results. However, individually examining the relationship between various factors and body image, it can be stated that our findings have been substantiated^3,13,28–33^.

Body mass index emerged as the strongest predictor of body image in the multivariable model. This finding aligns with previous research indicating that higher BMI is often associated with internalization of sociocultural appearance ideals and increased body dissatisfaction, particularly among women^6,7^. Importantly, this relationship may reflect not only physical characteristics but also weight-related stigma and self-objectification processes.

Rather than acting as isolated determinants, premenstrual symptom severity and poor sleep quality may jointly contribute to body image disturbances. The positive correlation observed between SQS scores and premenstrual symptom severity suggests that women reporting more severe PMS symptoms also tended to experience poorer subjective sleep quality, which is consistent with previous findings linking PMS with sleep disturbances^14,15^. This association is clinically plausible, as premenstrual symptoms frequently include pain, mood disturbance, irritability, bloating, and heightened physiological discomfort, all of which may interfere with sleep initiation and sleep continuity^14^. Conversely, poor sleep may exacerbate emotional reactivity, fatigue, and pain sensitivity, thereby intensifying the perceived burden of premenstrual symptoms^15,21^. Although causality cannot be inferred from the present cross-sectional design, the observed association highlights the close interrelationship between menstrual health and sleep in reproductive-aged women. PMS-related physical discomfort and affective symptoms, combined with impaired sleep, may reduce emotional regulation and body-related self-compassion, thereby increasing vulnerability to negative body evaluations^14,15^. This integrative interpretation helps explain why both variables independently predicted body image in the regression model.

To better understand the pathways through which these factors may influence body image, the relationship between premenstrual symptoms and physical activity was also examined. Our findings regarding the relationship between physical activity, PMS, and average weekday sleep duration revealed a negative correlation among the factors. This suggests that the more moderate work physical activity someone engages in, the more severe their premenstrual symptoms may be, and the less time they spend sleeping on weekdays. A negative correlation is also evident among vigorous work, sleep quality, and average weekday sleep duration. These results can be explained from two perspectives.

Examining the relationship between PMS and physical activity, it can be seen that moderate work physical activity negatively influenced the severity of PMS symptoms. Our findings are supported by the results of a previous study, which aimed to identify factors potentially explaining PMS using a multivariable linear regression model. Their results identified factors, including physical activity, as negatively influencing and exacerbating symptoms. Based on these findings, our own results are corroborated^34^. However, our own results also indicated a positive correlation between moderate recreation and PMS symptoms. These findings suggest a tendency that the more recreational physical activity one engages in, the milder their premenstrual symptoms may be. Thus, it is conceivable that while physical activity has been shown to have a positive impact on premenstrual symptoms according to numerous research findings^35–38^, the quality of physical activity (related to work, travel, or recreation), its intensity, and the balance of sleep duration can greatly determine the outcome of symptom changes.

Examining the relationship between sleep quality and physical activity, our results indicated that moderate work physical activity negatively impacts sleep quality. These findings were further supported by a randomized controlled trial, which showed that intensive physical activity may lead to a decrease in the time spent in the Rapid Eye Movement (REM) phase of sleep^39^. However, based on our own results, it can be assumed that there is a negative correlation between recreational physical activity and sleep quality. This correlation suggests a trend indicating that recreational physical activity may improve subjective sleep quality. Numerous scholarly articles report on the beneficial effects of physical activity on sleep quality^40–42^. However, in this case, the idea arises that the quantity and quality of physical activity may play a significant role in its exerted effects on sleep. However, it has to be emphasised that the study design does not allow for causal inferences.

The differential associations observed between work-related and recreational physical activity underscore the importance of activity context. While recreational physical activity is typically self-determined and linked to psychological benefits, work-related physical activity may be experienced as obligatory and physically demanding, potentially contributing to fatigue and reduced wellbeing^43,44^. These qualitative differences may partly explain their contrasting relationships with body image, sleep quality and premenstrual symptoms. The importance of the qualitative differences in physical activity is supported by numerous scholarly articles. Feig et al.’ research findings indicated that leisure-time physical activity showed a significant negative correlation with the occurrence of diabetes, obesity, and cardiovascular diseases, while such correlation was not observed with work-related physical activity^43^. Furthermore, the conclusion of White et al.’ research asserts that work-related physical activity cannot be promoted as compared to leisure-time physical activity^44^.

Based on our findings, participants engaged in more work-related physical activity on average than recreational physical activity. They spent at least 7 h sleeping on workdays on average, and they also spent more than 7 h sitting per day on average. According to the 24-Hour Movement Guidelines for Adults (18–64 years) and the American Physical Activity Guidelines, it is recommended to engage in at least 150 min of moderate to vigorous aerobic physical activity weekly^45^; U.S. Department of Health and Human Services^46^,. Based on our findings, this criterion was fulfilled, as participants engaged in an average of 532.10 ± 802.87 min per week of moderate and vigorous work-related physical activity, as well as 206.00 ± 455.44 min per week of moderate and vigorous recreational physical activity. According to the recommendations regarding sleep duration, 7–9 h of high-quality, regular sleep at regular intervals are necessary to exert its beneficial effects on health^45^. Based on our sample, we found that individuals spent 7.01 ± 0.92 h/day on weekdays and 8.39 ± 1.17 h/day on weekends sleeping, with an average of 7.69 ± 0.87 h/day of sleep, which aligns with the recommendations outlined in the guidelines. Finally, according to recommendations regarding sedentary time (sitting), it is advisable to limit daily sedentary time to 8 h or less and frequently interrupt prolonged periods of sedentary behaviour with standing breaks and other light-intensity physical activities^45^. Upon examining our own data regarding sedentary time, we observed that individuals spent an average of 7.17 h/day sitting, which also aligns with the recommendations outlined.

In summary, body image in reproductive-aged women was associated with a constellation of lifestyle and biological factors, including sedentary time, BMI, premenstrual symptom severity and sleep quality. These findings emphasise that promoting positive body image may require integrated strategies that address movement behaviours, sleep and menstrual health alongside weight-related factors. To our knowledge, this is the first study conducted in Hungary to examine the combined associations of movement behaviours, premenstrual symptom severity, sleep quality, and BMI with body image among reproductive-aged women. This context-specific contribution may support future women’s health research in Central and Eastern European populations.

## Limitations

A fact that can be considered a weakness of the study is that objective measuring instruments were not utilised. In the future, it is recommended to utilise accelerometers for monitoring physical activity instead of self-reported questionnaires. This approach will help to objectify our results. The same can be said for the assessment of sleep quality. Instead of self-reported questionnaires, it is recommended to employ polysomnography in future investigations and compare the obtained objective parameters. This approach will enhance the validity and reliability of the findings regarding sleep quality. In addition, body mass index was calculated from self-reported height and weight rather than direct anthropometric measurement. Self-reported BMI may systematically underestimate true BMI due to the tendency to over-report height and under-report weight, particularly among women, which should be considered when interpreting the observed associations^47^. Furthermore, another limitation relates to the assessment of premenstrual symptoms. Although the PAF-SF asks participants to reflect on symptoms experienced across the previous three menstrual cycles, the menstrual cycle phase at the time of questionnaire completion was not recorded or controlled for. Because symptom perception may vary according to current cycle phase, this may have introduced additional measurement variability into the reported PMS scores. Also, an additional limitation is that a priori sample size calculation was not performed before data collection. Although the final sample exceeded the minimum number estimated in a post hoc power analysis, the lack of prospective sample size planning should be acknowledged when interpreting the findings. While the achieved sample size was sufficient for the planned regression analyses, the sample may still be considered modest in relation to population representativeness and subgroup analyses. Therefore, larger and more diverse samples would be valuable in future studies to improve generalisability and enable more detailed stratified analyses. Additional limitations of the research include recruitment via social media platforms, which may carry the risk of potential bias. Although more complex analytical approaches such as structural equation modeling could further elucidate causal pathways among the examined variables, the cross-sectional and exploratory nature of the present study did not allow for robust causal modeling. Future longitudinal studies with larger samples are warranted to test potential mediation and directional effects.

## Data Availability

The datasets used and/or analysed during the current study are available from the corresponding author on reasonable request.

## Abbreviations

BAS: Body Appreciation Scale
BMI: Body Mass-Index
CHERRIES: Checklist for Reporting Results of Internet E-Surveys
GPAQ: Global Physical Activity Questionnaire
PAF-SF: Premenstrual Assessment Form-Short Form
PMS: Premenstrual Syndrome
REM: Rapid Eye Movements
SAGER: Sex and Gender Equity in Research
SPSS: Statistical Package for Social Sciences
SQS: Sleep Quality Scale
WHO: World Health Organisation
